# Classification of transient behaviours in a time-dependent toggle switch model

**DOI:** 10.1186/1752-0509-8-43

**Published:** 2014-04-04

**Authors:** Berta Verd, Anton Crombach, Johannes Jaeger

**Affiliations:** 1 EMBL/CRG Research Unit in Systems Biology, Centre for Genomic Regulation (CRG), Barcelona, Spain; 2, Universitat Pompeu Fabra (UPF), Barcelona, Spain

## Abstract

**Background:**

Waddington’s epigenetic landscape is an intuitive metaphor for the developmental and evolutionary potential of biological regulatory processes. It emphasises time-dependence and transient behaviour. Nowadays, we can derive this landscape by modelling a specific regulatory network as a dynamical system and calculating its so-called potential surface. In this sense, potential surfaces are the mathematical equivalent of the Waddingtonian landscape metaphor. In order to fully capture the time-dependent (non-autonomous) transient behaviour of biological processes, we must be able to characterise potential landscapes and how they change over time. However, currently available mathematical tools focus on the asymptotic (steady-state) behaviour of autonomous dynamical systems, which restricts how biological systems are studied.

**Results:**

We present a pragmatic first step towards a methodology for dealing with transient behaviours in non-autonomous systems. We propose a classification scheme for different kinds of such dynamics based on the simulation of a simple genetic toggle-switch model with time-variable parameters. For this low-dimensional system, we can calculate and explicitly visualise numerical approximations to the potential landscape. Focussing on transient dynamics in non-autonomous systems reveals a range of interesting and biologically relevant behaviours that would be missed in steady-state analyses of autonomous systems. Our simulation-based approach allows us to identify four qualitatively different kinds of dynamics: transitions, pursuits, and two kinds of captures. We describe these in detail, and illustrate the usefulness of our classification scheme by providing a number of examples that demonstrate how it can be employed to gain specific mechanistic insights into the dynamics of gene regulation.

**Conclusions:**

The practical aim of our proposed classification scheme is to make the analysis of explicitly time-dependent transient behaviour tractable, and to encourage the wider use of non-autonomous models in systems biology. Our method is applicable to a large class of biological processes.

## Background

Development in wild-type organisms is robust to genetic and environmental variations. Conrad Hal Waddington introduced the notion of ‘canalisation’ to describe how developmental processes resist perturbations during embryogenesis [1-3]. The canalised nature of development explains, he argued, why most phenotypes are discrete and distinct. To illustrate these ideas, he developed the epigenetic landscape, one of his most well-known concepts [3,4].

**Figure 1:**
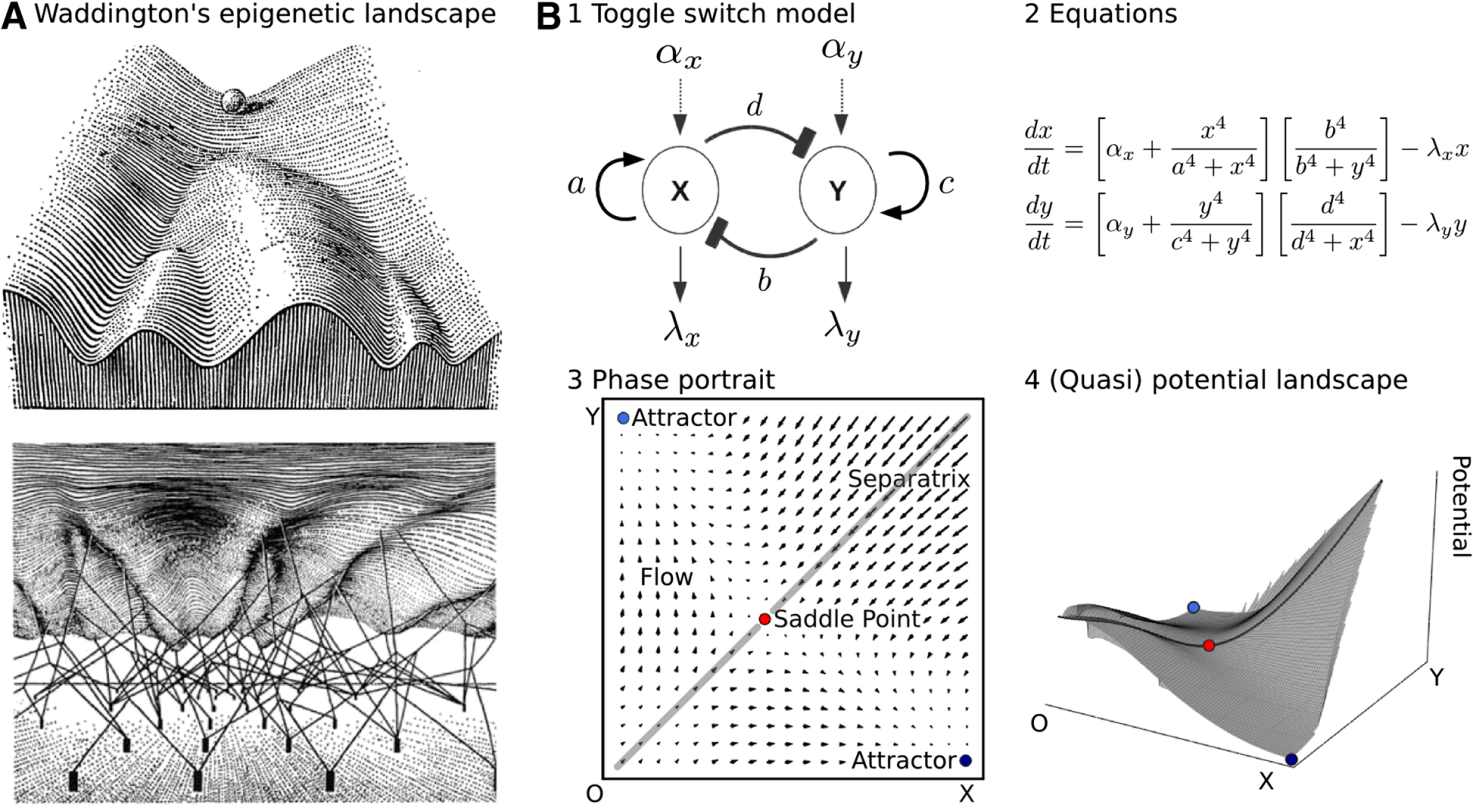
**Waddington’s epigenetic landscape and potential surfaces.(A)** Two different views of Waddington’s epigenetic landscape taken from “The Strategy of the Genes” published in 1957 [3]. The top panel shows a top view of the landscape. The path that the ball will follow represents the developmental trajectory (chreode) of a given system. Valleys indicate alternative differentiation pathways, branch points imply developmental decisions. The bottom panel shows the view from below the landscape. It illustrates how genes remodel the surface by pulling on it through ropes. Waddington used this sketch to show how the landscape’s topography changes during development and evolution. **(B)** Panel 1: Diagrammatic representation of the toggle switch network used in the simulations. Activating interactions are indicated by arrows, repressing ones by T-bar connectors. See Model and methods section for detailed parameter descriptions. Panel 2: Mathematical formulation of the toggle switch model. *x* and *y* indicate concentrations of the protein products of genes *X* and *Y*. Ordinary differential equations define the rate of change in protein concentrations ($\frac{\text{dx}}{\text{dt}}$ and $\frac{\text{dy}}{\text{dt}}$). Sigmoid functions with fixed Hill coefficients of 4 are used to represent auto-activation and mutual repression. Decay and external activation are taken to be linear. Parameters as in Panel 1. Panel 3: Phase portrait for a constant set of parameter values of the toggle switch model in the bistable regime. *X*- and *Y*-axes represent protein concentrations *x* and *y*. We use this example to illustrate relevant features of phase space: arrows indicate flow, blue points mark the position of stable steady states (attractors), the red point shows an unstable steady state (saddle) lying on the separatrix that divides the two basins of attraction (grey line). See the main text for detailed descriptions of the highlighted features. Panel 4: Quasi-potential landscape associated to the the phase portrait shown in Panel 3. The steepness of the quasi-potential surface correlates with the flow at each corresponding point on the phase portrait. Attractors, saddle, and separatrix are indicated as in Panel 3. See main text for details.

In Waddington’s epigenetic landscape, the current state of a developing system is indicated by a ball on an undulated surface (Figure 1A, top panel) [3,5,6]. The topography of this landscape determines the developmental potential or repertoire of the system. The top-most edge of the surface shown in Figure 1A (top panel) represents the initial state of the system given by, for example, a particular set of initial protein concentrations in a cell. Valleys in the landscape symbolise the various differentiation pathways that are available. The landscape’s topography—together with the initial state—determine a developmental trajectory that follows a particular valley. The structure of the landscape is such that, if the system is slightly perturbed, the sloping valley walls will cause it to correct and readjust its trajectory. This behavour is called ‘homeorhesis’—the maintenance of a dynamic trajectory—in analogy to the more static concept of homeostasis—the maintenance of a (steady) state of the system [3]. The wider and deeper a valley is, the more canalised the developmental trajectory. Waddington named such canalised trajectories ‘chreodes’.

An additional feature of Waddington’s landscape is crucial in our context: it is the fact that its surface is not necessarily fixed, but can change over time due to the influence of genetic or environmental signals. Waddington represented this by pegs connected to the underside of the landscape by ropes (Figure 1A, lower panel) [3]. As the genetic or environmental context of the system changes, these ropes pull and stretch the surface, thereby changing its topography and hence its developmental potential.

Waddington’s epigenetic landscape was conceived as a conceptual tool to illustrate the nature of developmental robustness and its effect on evolutionary dynamics [1,3-5,7]. As such, it remained at a rather metaphorical level of explanation, since complex non-linear biological processes are hard to formulate and analyse [5,6,8-12]. This is still the predominant way in which it is used in various reviews on contemporary stem cell research (see for example, [13-17]). However, in order to understand the precise nature of specific developmental trajectories or chreodes in real systems, we have to take Waddington’s landscape a step further: we have to calculate it based on experimental evidence, and use it to characterise the transient behaviours that govern the observed developmental dynamics [18-25].

The increasing availability of quantitative gene expression data renders this approach feasible. However, before we can successfully apply it, we also require new conceptual and mathematical tools to deal with the analysis of data-driven models that are formulated in terms of non-autonomous (i. e. explicitly time-dependent) dynamical systems. Explicit time-dependence is necessary to reproduce the changing topography of Waddington’s landscape. However, such systems are difficult to study in a rigorous mathematical manner, and few analysis tools exist at this point. In this work, we address this challenge by proposing a classification scheme for transient dynamic behaviours observed in a non-autonomous version of a simple gene network model. This scheme is meant to provide the foundation for the analysis of more complex time-dependent models that reproduce the dynamics of specific, experimentally tractable, biological regulatory systems.

### Dynamical systems

In this study, we focus on dynamical systems formulated in terms of ordinary differential equations, and illustrate how such models can help explain the function and potential of developmental gene regulatory networks in terms of their dynamical repertoires, that is, in terms of the set of behaviours that can be implemented by the system. A system’s behaviour is defined by its trajectories, which represent the change of the state of the system—e. g. consisting of a set of transcription factor concentrations—over time. The shape of these trajectories depends on the structure or organisation of the underlying regulatory network (see, for example, [3,22-24,26-31]). It is possible to gain a general qualitative understanding of what the system’s trajectories can and cannot achieve without the need to solve the dynamical system analytically [32,33]. Most of this information comes from the geometrical analysis of phase space, i. e. an analysis of the number, nature and relative arrangement of the steady states of the system.

The phase space of a dynamical system is an abstract space, in which each dimension represents the value of a specific state variable. Here we use a well established double-repressive feedback loop model, known as the toggle switch ([23], and references therein), to study transient dynamics in a two-dimensional, time-dependent gene regulatory network (see Figure 1B, panels 1 and 2, as well as ‘Model and methods’ below, for the full formulation). In this case, the state variables represent the concentrations of the transcription factors that constitute the network (denoted by *X* and *Y*; Figure 1B, panel 1). The graphical representation of phase space is called the phase portrait. It shows the rate of change of the system at any given state. This is known as the flow of the system. The flow of the toggle switch model is indicated by arrows of a given length and direction in the phase portrait shown in Figure 1B, panel 3. If we follow the flow from all possible initial states, we obtain the totality of possible dynamic trajectories.

It is evident from the inspection of the flow in Figure 1B (panel 3) that trajectories tend to converge to specific points in phase space: the steady states of the system. There are different kinds of steady states, those that are stable, and those that are unstable. The most simple stable steady state is an attractor point [32,33]. Attractors, as their name implies, draw trajectories towards them. Furthermore, they have the special property that once a trajectory has reached an attractor, it will return to it if the system is slightly perturbed. An example of an unstable steady state is a saddle point (Figure 1B, panel 3). Saddle points attract trajectories from some directions, but repel them in others. Usually, the system will move away from a saddle upon perturbation, towards the nearest attractor. The repelling trajectory follows a structure in phase space called an unstable manifold. This manifold is defined by the the path that links a saddle with an attractor point. Note that unstable manifolds correspond to chreodes at the bottom of valleys in Waddington’s landscape.

In our phase portraits, we plot steady states as points coloured according to their stability: attractors in blue, and saddle points in red (Figure 1B, panel 3). The region of phase space around an attractor, from which all trajectories converge towards it, is called its basin of attraction. Curves known as separatrices set apart the different basins and their attractors (in the case of Figure 1B, panel 3, the separatrix is a straight line indicated in grey). Saddle points are always located on separatrices.

Attractors and saddles, with their associated basins and separatrices can be created or annihilated through the process of bifurcation [32,34]. Bifurcations represent sudden qualitative changes in the structure of the phase portrait caused by small changes in the values of a given set of control parameters.

To date, the best example illustrating the importance and usefulness of geometric approaches for understanding the dynamics and function of specific, experimentally tractable, developmental regulatory systems comes from an analysis of the gap gene regulatory network involved in pattern formation during early embryogenesis of the vinegar fly *Drosophila melanogaster*. Manu and colleagues [35] identified features of phase space responsible for patterning and canalisation of spatio-temporal gene expression in the *Drosophila* blastoderm embryo. The analysis is based on low-dimensional projections of phase space to study the geometric arrangement of attractors in the four-dimensional system representing the change in protein concentration for four transcription factors encoded by the gap genes *hunchback (hb)*, *Krüppel (Kr)*, *knirps (kni)*, and *giant (gt)*. Gene expression domain boundaries that remain at a constant position over time could be attributed to movements of attractors in phase space, or nuclei switching between attractor basins depending on their position in the embryo. In contrast, more posterior expression boundaries, which keep shifting position over time, were associated with transient behaviours along a canalising unstable manifold, forming the equivalent of a valley in Waddington’s landscape [35].

### Potential landscapes

Phase portraits of systems with two state variables can be visualised in terms of their associated potential landscape [32] (Figure 1B, panel 4). In this representation, the steepness of the potential landscape corresponds to the flow of the system (compare Figure 1B, panels 3 and 4). Trajectories travel downhill towards the attractor points. The topography of the potential landscape is therefore a direct result of the topology (number and nature of steady states) and the geometry (relative positions of the steady states and size of the flow) of the underlying phase portrait.

Potential surfaces can be thought of as mathematical representations of Waddington’s epigenetic landscape [18,20,24,25,36-40]. Attractors represent differentiated states (in agreement with earlier postulates, [1,13,14,41-46]), separatrices form the ridges between the valleys, which are formed by unstable manifolds representing their associated chreodes (see Figure 1B, panels 3 and 4), and the flow is represented by the steepness of the slopes on the landscape. Canalisation is explained by the depth and width of each valley in the potential landscape. This provides a way of probing and understanding the features that confer robustness to the system (see, for example, [18,19,21,24,45,47,48]). Branching valleys can arise through particular types of bifurcation events. In particular, new attractor states can branch off from existing ones during development or stem cell differentiation through (supracritical) pitchfork bifurcations [19,20,25,32,47,49-51].

Due to this analogy to Waddington’s epigenetic landscape, potential landscapes are becoming increasingly popular as explanatory tools in fields such as evo-devo, developmental biology, and especially in stem cell research. In the case of stem cells, the positioning of the valleys in the landscape relative to each other explains which differentiation pathways can be reached by a given stem cell, and which cell fates can (or cannot) be trans-differentiated into each other [13-16,36,37,40,52-54]. This illustrates how specific biological meaning can be gained from studying the topography of potential landscapes, which ultimately, is nothing more than a visually accessible way of studying the underlying phase portrait.

Waddington meant his epigenetic landscape as a “diagrammatic representation” of development and warned explicitly against interpretations that were too rigorous or literal [2,3]. Detailed topographical interpretations of Waddington’s landscape may be quite inaccurate and even misleading. Ferrell [25] points out that Waddington’s landscape, where valleys progressively split into an increasing number of branches, does not help explain many realistic cell differentiation processes. In particular, many inductive processes in development (e. g. vulval induction in the roundworm *Caenorhabditis elegans*, or mesoderm induction in vertebrates) involve saddle-node rather than pitchfork bifurcations, which correspond to the disappearance of valleys rather than to their creation by branching. The system shifts to a new attractor only once the old one has vanished. This is why it is important to move from metaphorical uses of Waddington’s epigenetic landscape to accurately calculated potential surfaces whenever possible, or more importantly, to the detailed analysis of the underlying phase portraits, which is where the dynamics of specific regulatory networks are determined.

Potential landscapes can only be calculated and visualised explicitly for a restricted range of dynamical systems, belonging to the class of gradient systems [32]. Note that the notion of a gradient system is defined mathematically by the absence of limit cycles or any other complex attractor structures in their phase portraits, and has nothing to do with biochemical or other biological gradients (see Model and methods for a detailed explanation). In cases where we do not know whether the system under study is a gradient system or not, we can approximate the actual potential using various numerical methods [19,20,38,39,48,55]. The resulting approximations are called quasi-potential landscapes.

However, even such quasi-potential landscapes can only be visualised directly when the number of state variables of the system does not exceed two. This is not true for most biologically realistic systems. Nevertheless, (quasi-)potential landscapes are still useful as conceptual tools for the analysis of higher-dimensional regulatory networks. In some cases, it is possible to reduce a high-dimensional system to a lower-dimensional one (see, for example, [56-58]). But even if this is not the case, the concept of the potential landscape provides two advantages. First, as discussed above, quasi-potential surfaces link Waddington’s intuitively accessible concept of the epigenetic landscape to the biological interpretation of (high-dimensional) phase space analysis. And second, potential surfaces are useful as visual guides to diagnose features of the underlying phase portrait of the system that are characteristic of specific dynamic behaviours of the regulatory network under study.

### The importance of transient dynamics

Many models of biological systems are formulated with the assumption that the relevant dynamics occur near or at a steady state. For instance, Thom’s pioneering systematic and rigorous analysis of morphogenesis in terms of catastrophe theory explicitly and strongly relies on this assumption [8]. Similarly, work on robustness and evolvability, using ensembles of simulated networks, standardly assumes that the steady state pattern produced by a model can be taken as a satisfactory and realistic representation of the phenotype of the system (e. g. [59-62]). Furthermore, stem cell models that make use of potential landscapes are analysed with a strong focus on how their attractors govern system behaviour [19,20,47,48,56,58].

In some cases, the steady state assumption makes obvious sense based on biological reasoning. One example is the analysis of the segment polarity network in *Drosophila melanogaster*, which amplifies and maintains a periodic input, and thus performs an intrinsically stabilising patterning function [63-65]. In most cases, however, biological pattern formation is highly dynamic and far from equilibrium, and the steady state assumption is justified based on methodological, rather than biological considerations. Focussing on asymptotic behaviour at or near steady state greatly simplifies the analysis of the system. First, it discretises and reduces phase (and hence phenotype) space into a small number of possible states—represented by the system’s attractors (see Figure 1C, panel 3). Second, it enables the powerful toolkit of linear stability analysis to be employed to examine the characteristic properties of system states [32,33].

However, there are both theoretical and practical reasons indicating that steady state analysis misses essential and biologically relevant systems behaviours. One line of theoretical reasoning is provided by Waddington himself, who reminds us that “[i]n the study of development we are interested not only in the final state to which the system arrives, but also in the course by which it gets there”. [3]. His concept of homeorhesis and his representation of developmental trajectories or chreodes as descending valleys in the epigenetic landscape places the focus explicitly on the transient dynamics of cells on their way to their final, differentiated state. In the same spirit, other authors have suggested that phenotypes should be defined over developmental trajectories, rather than representing some sort of ‘final’ outcome or ‘end state’ of the system [22,66]. To decide what a final pattern is, and when exactly it occurs, is always arbitrary to some degree, while transient features (such as intermediate stages and the timing of their transitions) are clearly important when considering the function and dynamics of developmental processes.

There are also practical reasons to consider transient dynamics explicitly. For developmental processes that consist of continuous transitions between patterns rather than the production of a final output, it is impossible to decide *a priori* whether the system is representing a non-autonomous succession of steady states (see below), or whether its behaviour is truly transient (i. e. far from steady state). In the case of gap domain shifts, we have evidence for the latter [35], although there is no reason to assume that the two situations need to be mutually exclusive. The gap gene model analysed by Manu and colleagues [35] exhibits boundary shifts that are caused by trajectories following a canalising unstable manifold. Assuming steady state dynamics would collapse the trajectories representing these shifts into a single attractor point at the final configuration of gene expression. Any such analysis would miss the relevant underlying features of phase space (the transient manifold), and therefore fail to provide a proper characterisation and explanation for the observed gene expression dynamics (the shift in domain position over time).

Other examples of developmental processes, where transient dynamics are clearly important are dorso-ventral patterning of the vertebrate neural tube, which involves boundary shifts strikingly similar to those of the gap domains [67,68], and vertebrate somitogenesis or short-germband segmentation in arthropods where transient travelling waves of gene expression are an essential component of the underlying clock-and-wavefront patterning mechanism [69-75]. Incidentally, similar considerations can be made for models dealing with ecological networks, and several examples exist in the literature that consider transient dynamics explicitly (see models of coupled oscillating population dynamics between species [76,77], or [78-80]).

### Non-autonomy: explicit time dependence

Another important aspect of biological regulatory processes, which receives surprisingly little attention, is the explicit time dependence of these systems. As soon as we consider cellular dynamics, development, or evolution over a large-enough time span, the organisation of the underlying regulatory system starts to change. This affects the parameters—not just the state variables—of the system. Such explicitly time-dependent dynamical systems are called non-autonomous [32,81,82]. Time-dependent signalling cues and environmental conditions have long been known to shape many processes in the fields of evolutionary and developmental biology. Obvious examples of such phenomena are inductive processes or external (e. g. seasonal) cues that are essential to trigger many developmental pathways (as described in standard textbooks such as [83,84]), or evolutionary dynamics driven by changing environmental conditions (examples, based on the simulation of gene regulatory network models, can be found in [85-88]).

Still, it is rare to find studies based on explicitly non-autonomous models in the literature, and most authors avoid the challenge of dealing with dynamical systems where the parameters representing external cues are time-dependent. This is the case in the study of gap domain shifts by Manu and colleagues [35,89], where maternal morphogen gradients providing regulatory input to the system were assumed to reach steady state before gap gene boundary positioning was analysed. Such simplifications can be risky, especially when describing biological phenomena where the time scales of change in parameters and state variables are of similar order. In such cases, time scales should not be separated, nor quasi-steady states considered since it is easy for dynamical properties and behaviours of the system to be missed or misinterpreted under these conditions.

Recently there have been some attempts at including non-autonomy in biological models. Corson and Siggia [90], for example, offer an explanation for vulva development in *C. elegans* which explicitly considers temporal parameter changes due to inductive signalling cues in their model. Their model considers differentiation of cells into three differentiated states depending on inputs from two signalling pathways. Signalling inputs are encoded in the model by altering values of system parameters, which change and distort the geometry of the phase portrait by displacing separatrices from their original position (see Figures three–nine in [90]). When a cell receives a signal, its developmental trajectory comes to lie within another basin of attraction, inducing an alternative cell fate to that which would have been reached in the absence of the signal. This study illustrates the importance of non-autonomous dynamics in development. However, it remains somewhat limited in its implementation of explicit time-dependence. Although parameter change is included in the model, signalling occurs before cells embark on their developmental paths, and trajectories develop purely autonomously thereafter. Therefore, this approach does not fully capture the transient dynamics of cell differentiation. In other words, although their model is able to offer an explanation for cell differentiation in vulval development, it cannot capture the full non-autonomous nature of the developmental process, since it does not reproduce developmental trajectories, chreodes, in an accurate and fully time-dependent manner.

Similarly, most of the few other examples of non-autonomous models in the biological literature do not explicitly consider the effect of parameter changes on transient behaviour (e. g. [91-93]). This simplification may be justified in many cases and is necessary for any kind of rigorous analytical treatment of a model. In many situations, however, it fails to capture essential features of the system. For instance, a truly accurate analysis of gap gene regulatory dynamics would require the inclusion of both non-autonomy from the rapidly changing maternal morphogen gradients, and transient dynamics, which are known to underlie the temporal shifts in domain position. Before we can undertake such an analysis we must first build a conceptual toolkit for phase space analysis of transient, non-autonomous dynamics. Due to the limited amount of analysis possible in such systems, this toolkit will need to be developed in a pragmatic and empirical manner, using numerical simulation and exploration of a simple conceptual model as its basis.

In the following sections, we present such a simulation-based attempt at developing concepts to classify transient, non-autonomous behaviours. For this purpose, we use a simple two-component model of a genetic toggle switch, whose potential landscape can be explicitly visualised. We use time series of graphs and animations of systems dynamics on this potential to identify mechanisms leading to state transitions, and other forms of pattern formation. From this, we are able to identify four basic types of dynamical mechanism and behaviour—transitions, pursuits and two types of captures of trajectories—that can be used to classify and understand dynamical behaviour in more complicated and realistic models, such as a full non-autonomous version of the gap gene model. While this present paper provides the methodological foundation for such an analysis, detailed results for a realistic model of the gap genes will be presented elsewhere.

## Model and methods

To develop our methodology for analysing transient behaviour in non-autonomous dynamical systems, we use a simple toggle switch model (see [22], and references therein) with time-dependent parameters. We consider two interacting genes *X* and *Y* (Figure 1B, panel 1). Concentrations of the corresponding protein products are labelled *x* and *y*. *X* and *Y* mutually repress each other, are linearly activated by external signals and can auto-activate themselves (Figure 1B, panel 1). Protein products decay linearly dependent on their concentration. The mathematical formulation of our toggle switch model is thus given by

(1) $$\begin{array}{lcrr} \frac{\text{dx}}{\text{dt}} & = & \left[\alpha_{x}+\frac{x^{4}}{a^{4}+x^{4}}\right]\left[\frac{b^{4}}{b^{4}+y^{4}}\right]-\lambda_{x}x & \\ \frac{\text{dy}}{\text{dt}} & = & \left[\alpha_{y}+\frac{y^{4}}{c^{4}+y^{4}}\right]\left[\frac{d^{4}}{d^{4}+x^{4}}\right]-\lambda_{y}y & \end{array}$$

where parameters *α*_*x*_ and *α*_*y*_ represent the external activation on genes *X* and *Y* respectively. Sigmoid functions with Hill coefficients of 4 are used to represent auto-activation and mutual repression, where parameters *a* and *c* determine auto-activation thresholds, while *b* and *d* determine thresholds for mutual repression. Protein decay rates are represented by parameters *λ*_*x*_ and *λ*_*y*_.

**Figure 2:**
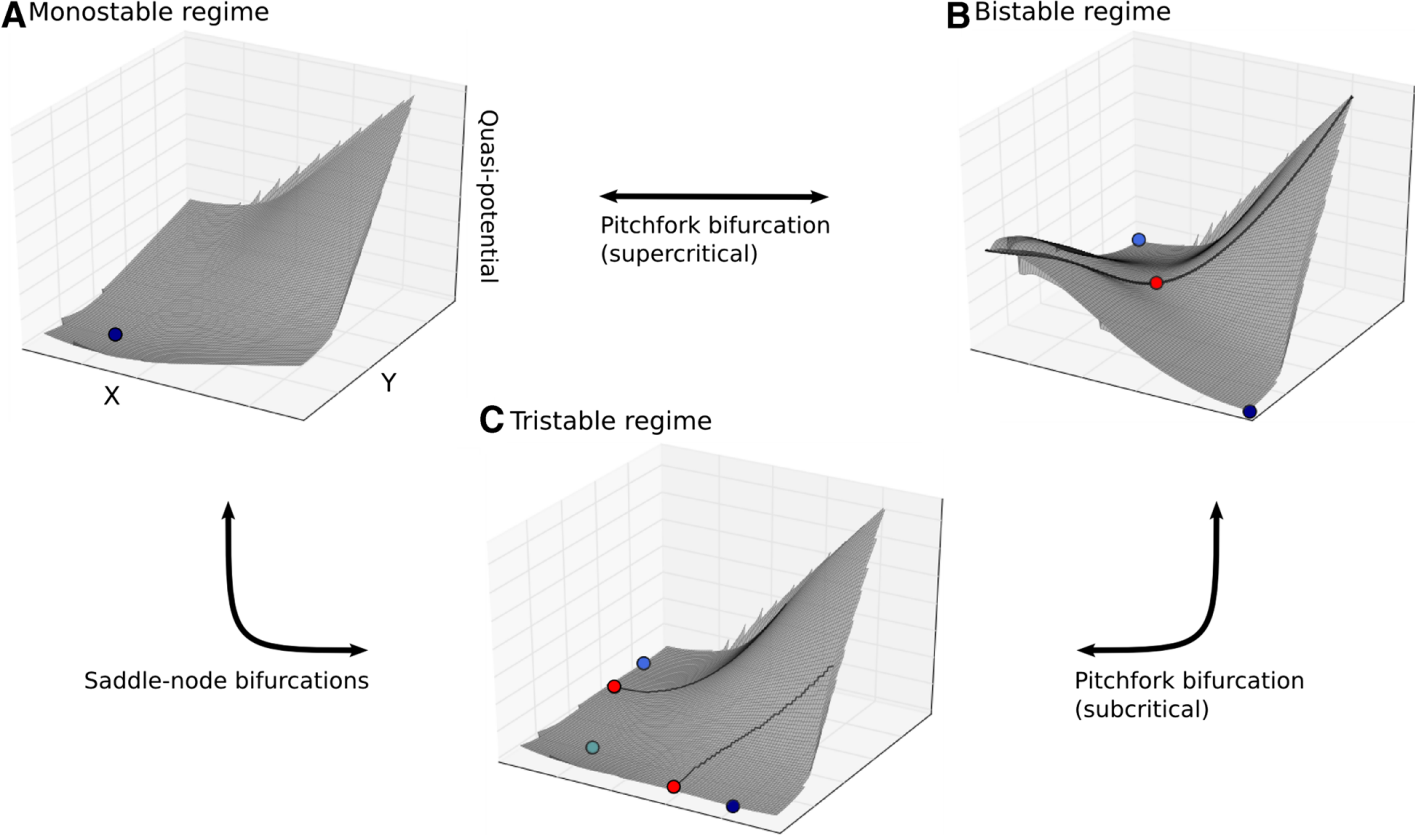
**Dynamical regimes of the toggle switch model.** The toggle switch model can exhibit three different dynamical regimes depending on parameter values. **(A)** In the monostable regime, the phase portrait has one attractor point only (represented by the blue dot on the quasi-potential landscape). At this attractor, both products of *X* and *Y* are present at low concentrations. **(B)** In the bistable regime, which gives the toggle switch its name, there are two attractor points (shown in different shades of blue) and one saddle (red) on a separatrix (black line), which separates the two basins of attraction. The attractors correspond to high *x*, low *y* (dark blue), or low *x*, high *y* (light blue). The two factors never coexist when equilibrium is reached in this regime. **(C)** In the tristable regime, both bistable switch attractors and the steady state at low co-existing concentrations are present (shown in different shades of blue). In addition, there are two separatrices with associated saddle points (red). These regimes convert into each other as follows (double-headed black arrows indicate reversibility of bifurcations): the monostable attractor is converted into two bistable attractors and a saddle point through a supercritical pitchfork bifurcation; the saddle in the bistable regime is converted into an attractor and two additional saddles in the tristable regime through a subcritical pitchfork bifurcation; the bistable attractors and their saddles collide and annihilate in two simultaneous saddle-node (or fold) bifurcations to turn the tristable regime into a monostable one. Graph axes as in Figure 1B, Panel 4.

The toggle switch model (1) exhibits different dynamical regimes depending on the values of its parameters (Figure 2A–C). Its name derives from the fact that it can exhibit bistability over a wide range of parameters. When in this bistable region of parameter space, the underlying phase portrait has two attracting states and one saddle point (Figure 2B). All phase portraits associated with parameters in the bistable range are topologically equivalent to each other, meaning that they can be mapped onto each other by a continuous deformation of phase space called a homeomorphism [34].

The toggle switch model has two other dynamical regimes: monostable and tristable. Phase portraits associated with parameters in the monostable range have only one attractor point (Figure 2A), while those in the tristable range have three attractor states and two saddle points (Figure 2C). Again, phase portraits within each regime are topologically equivalent to each other. While phase space can be geometrically deformed within each regime (through movements of attractors or separatrices), its topology only changes when one regime transitions into another through different types of bifurcations [32,34] (Figure 2). The transition from monostable to bistable is known to be governed by a supracritical pitchfork, the transition from bistable to tristable involves a subcritical pitchfork bifurcation, and the transition from tristable to monostable takes place through two simultaneous saddle-node bifurcations involving the two attractors labelled in darker blue in Figure 2C.

### Definition of the potential landscape

Potential landscapes can only be calculated explicitly for the class of dynamical systems called gradient systems [32]. A two-variable gradient system is a dynamical system

(2) $$\begin{array}{lcrr} \frac{\text{dx}}{\text{dt}} & = & f(x,y) & \\ \frac{\text{dy}}{\text{dt}} & = & g(x,y) & \end{array}$$

which satisfies the following relationship between partial derivatives

(3) $$\;f_{y}(x,y)=g_{x}(x,y).$$

For gradient systems, it is possible to calculate a closed-form (explicit) potential function, *V*(*x*,*y*) such that

(4) $$\begin{array}{lcrr} V_{x} & = & \frac{-\text{dx}}{\text{dt}} & \\ V_{y} & = & \frac{-\text{dy}}{\text{dt}}. & \end{array}$$

The local minima on the two-dimensional potential surface given by *V*(*x*,*y*) correspond mathematically to the steady states of the system in (2) since, if (*x*^∗^, *y*^∗^) is such that

(5) $$\;V_{x}(x^{*},y^{*})=V_{y}(x^{*},y^{*})=0,$$

then

(6) $$\begin{array}{lcrr} \frac{\text{dx}}{\text{dt}}(x^{*},y^{*}) & = & 0 & \\ \frac{\text{dy}}{\text{dt}}(x^{*},y^{*}) & = & 0, & \end{array}$$

and, therefore, (*x*^∗^, *y*^∗^) is a steady state of (2).

### Calculating quasi-potential landscapes

Condition (3) will not always be met. In particular, dynamical systems representing gene interaction networks are not in general gradient systems, and therefore an associated potential function and landscape may not exist. In such cases, we can still take advantage of the visualisation power of potential landscapes by approximating the true potential using a numerical method. The numerical approximation method we adopt for our study was developed by Bhattacharya and colleagues [38] using a toggle switch model very similar to the one used here. This allows us to calculate a quasi-potential landscape for any specific set of fixed parameter values.

The quasi-potential, which we denote by *V*_*q*_, is defined to decrease along all trajectories of the dynamical system as they progress on the phase portrait over time

(7) $$\Delta V_{q}=\frac{\delta V_{q}}{\mathrm{\delta x}}\mathrm{\Delta x}+\frac{\delta V_{q}}{\mathrm{\delta y}}\mathrm{\Delta y}=\frac{-\text{dx}}{\text{dt}}\mathrm{\Delta x}+\frac{-\text{dy}}{\text{dt}}\mathrm{\Delta y.}$$

*Δx* and *Δy* are defined as small-enough increments along the trajectory such that *dx*/*dt* and *dy*/*dt* can be considered constant in the interval [(*x*,*x*+*Δx*),(*y*,*y*+*Δy*)]. In addition, $\mathrm{\Delta x}=\frac{\text{dx}}{\text{dt}}\mathrm{\Delta t}$ and $\mathrm{\Delta y}=\frac{\text{dy}}{\text{dt}}\mathrm{\Delta t}$, where *Δt* is the time increment. Substituting into equation 7, we obtain

(8) $$\begin{array}{c} \Delta V_{q}=\frac{-\text{dx}}{\text{dt}}\left(\frac{\text{dx}}{\text{dt}}\mathrm{\Delta t}\right)+\frac{-\text{dy}}{\text{dt}}\left(\frac{\text{dy}}{\text{dt}}\mathrm{\Delta t}\right)\\ =-\left[{\left(\frac{\text{dx}}{\text{dt}}\right)}^{2}+{\left(\frac{\text{dx}}{\text{dt}}\right)}^{2}\right]\mathrm{\Delta t.} \end{array}$$

*ΔV*_*q*_ has been formulated in such a way that, for positive time increments *Δt*, *ΔV*_*q*_ is always negative along the unfolding trajectory and is, in effect, a Lyapunov function of the two-gene dynamical system [32]. This ensures that trajectories will always “roll” downhill on the quasi-potential surface. Just as in the case of closed-form potential, the steady states of the system (*x*^∗^,*y*^∗^) correspond to the local minima on the quasi-potential surface since *ΔV*_*q*_(*x*^∗^,*y*^∗^)=0.

We apply the numerical approximation method described above to trajectories with various initial points on the *x*-*y* plane. This yields a sampled collection of trajectories with quasi-potential values associated to every one of their points. Next, we apply the following two assumptions, in order to construct a continuous quasi-potential surface from this sample of discrete trajectories [38]: 

1. Two trajectories with different initial conditions that converge to the same steady state must also converge to the same final quasi-potential level (normalisation within basins of attraction).

2. Two adjacent trajectories that converge to different steady states will be taken to start from the same initial quasi-potential level (normalisation between basins of attraction).

Finally, interpolation of all the normalised trajectories results in a continuous quasi-potential landscape. Bhattacharya *et al.*[38] validated this approach by demonstrating that the quasi-potential values of the steady states were inversely correlated with their probability of occurrence using a stochastic version of the toggle switch dynamical system.

### Approximating non-autonomous trajectories

**Figure 3:**
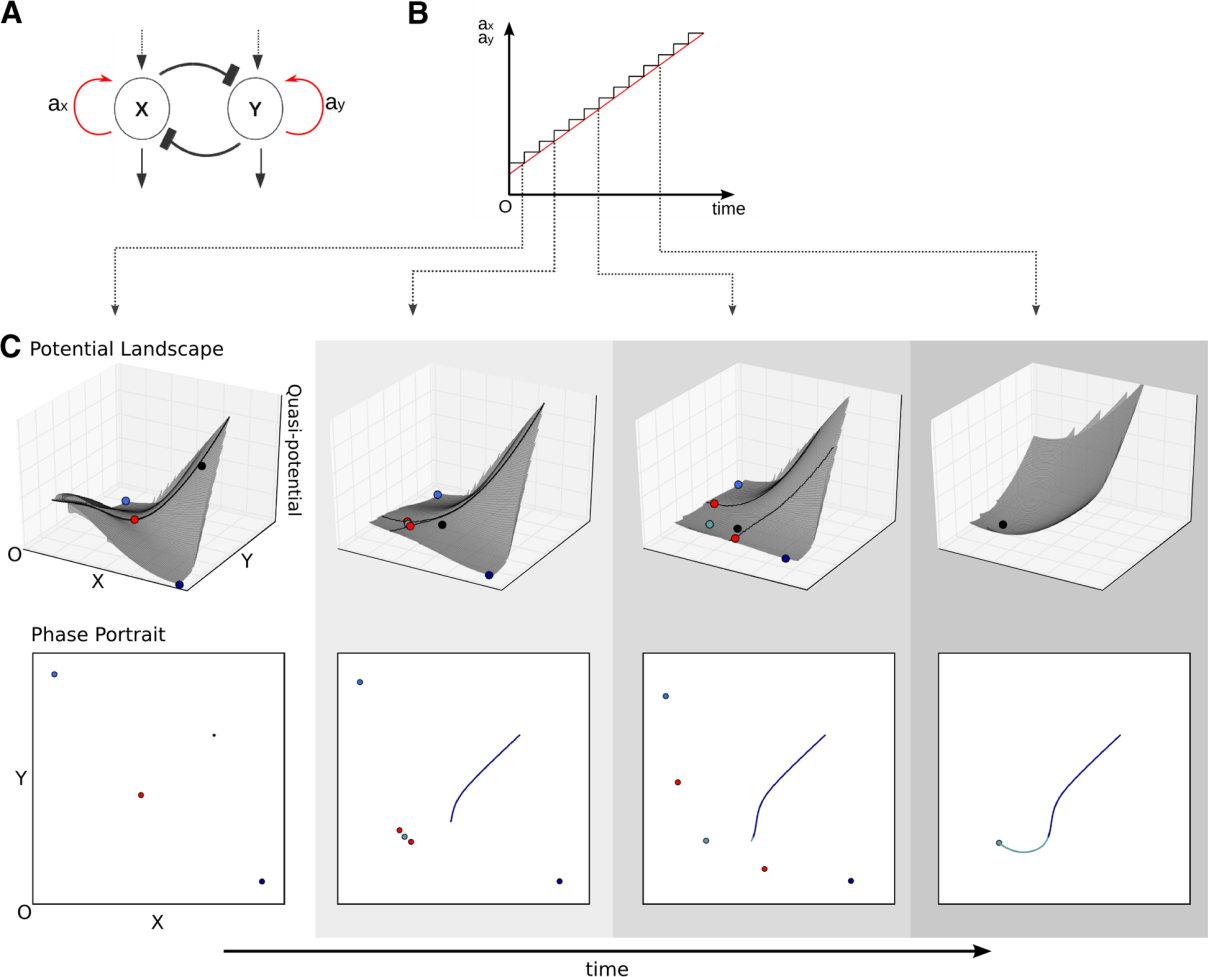
**Numerical approximation of non-autonomous trajectories.(A)** Toggle switch network. Red arrows representing auto-activation indicate time-dependence of threshold parameters *a*_*x*_ and *a*_*y*_ (see equation 1). **(B)** Values of auto-activation thresholds *a*_*x*_ and *a*_*y*_ are altered simultaneously and linearly over time. The graph shows the step-wise approximation of a continuous change, in this case, an increase in parameter values. Step size is taken as small as computational efficiency allows. **(C)** During every time step, parameters can be considered constant, and the phase portrait and (quasi-)potential landscape are calculated for the current set of parameter values. Trajectories are then integrated over the duration of the time step using the previous end point as the current initial condition. The result is mapped onto the potential surface. The four panels in (C) show examples of potential landscapes (upper panels) calculated based on sets of parameter values at time points indicated by dashed arrows from (B). Important events altering the geometry of the trajectory are indicated. Lower panels show the corresponding instantaneous phase portraits with the integrated progression of the trajectory across time steps. See Model and methods for details.

As we have argued in the Background Section, we cannot generally assume that parameter values remain constant over time when modelling biological processes. We take a step-wise approximation approach to the change in parameter values to address this problem (Figure 3). We chose a time increment (step size) as small as possible. Parameter values are kept constant for the duration of each time step. As a consequence, the associated phase portrait will also remain constant during this time interval, and is visualised for each step by calculating a quasi-potential landscape as described in the previous section (Figure 3C, top row).

The smaller the time increments considered, the better we are able to approximate continuous changes in parameter values, and the consequent changes to the associated phase portrait and quasi-potential landscape. Such accurate approximation allows us to faithfully reproduce non-autonomous trajectories produced by models with continuously time-variable parameters. This is done by integrating trajectories using constant parameters during each time step, and then using the resulting end position in phase space as the initial condition for the next time step. The resulting integrated trajectories can then be visualised by mapping them from the underlying phase plane onto the associated quasi-potential landscape as described above. This allows us to track and analyse in detail how changes in the phase portrait and quasi-potential landscape shape the trajectories as the values of the parameters are changing.

## Results and discussion

Using the toggle switch defined in (1) as a conceptual model, we have set out to identify and catalogue mechanisms that affect the shape of trajectories in non-autonomous systems. In contrast to previous studies (see, for example, [19,20]), we do not assume asymptotic behaviour of the system, but focus on transient dynamics—that is, the entire trajectory from initial to steady state.

In this context, we use the term ‘mechanism’ in an unusual, but precisely defined way. We do not mean it to imply any specific biochemical interactions—this is how most experimental biologists would use the term. Nor does our notion of ‘mechanism’ depend on any specific regulatory structure, such as network motifs (reviewed in [94]), or the different stripe-forming mechanisms studied in [95]. Instead, we use the term ‘mechanism’ in a broader, more abstract sense: a mechanism is a causal explanation of dynamics in terms of the dependence of the flow on parameter changes, and how these affect the trajectories of the system. We will illustrate this very abstract definition using a number of concrete examples below.

Changing parameter values in non-autonomous systems can change the underlying phase portrait in two main ways: it can alter the *geometry* or the *topology* of phase space (or both) [32,34]. On one hand, a change in geometry means that the phase portrait is still composed of the same basic elements—the same number, kind and relative placement of attractors and their associated basins, saddle points, and separatrices—but that the exact position, shape, and/or size of these elements has been altered. On the other hand, a topological alteration of phase space occurs when a change in parameter values cause the number or stability of steady states to change. For example, small changes in a parameter value can cause a pair of attractor and saddle points to appear or disappear through a saddle-node or fold bifurcation, or an attractor can be turned into a saddle point through a transcritical bifurcation. Topological changes to the phase portrait are therefore associated with bifurcations as studied in the context of autonomous dynamical systems [32-34].

In what follows, we examine how both geometrical and topological changes in phase space affect transient trajectories in non-autonomous systems. For this purpose, we use a numerical simulation approach with three-dimensional visualisation of the quasi-potential landscape associated with the phase portrait of our toggle switch model (see the Model and methods Section, for details). We identify four different basic types of non-autonomous mechanisms affecting dynamical behaviour: transitions, pursuits and two kinds of captures.

### Transitions

Out of the four trajectory-shaping mechanisms that we have identified in this study, transitions are the most familiar. They have been found and described in previous approaches to the study of non-autonomous biological systems [78-80,91,92]. Transitions in our context are equivalent to Thom’s ‘catastrophes’ [8], and to what Scheffer has termed ‘critical transitions’, defined as a shift of the system from one attractor to another when it passes a given critical point in parameter space [92]. While Thom’s and Scheffer’s analyses focus on the resulting steady state behaviour of the system, we also consider the transient dynamics during a transition.

**Figure 4:**
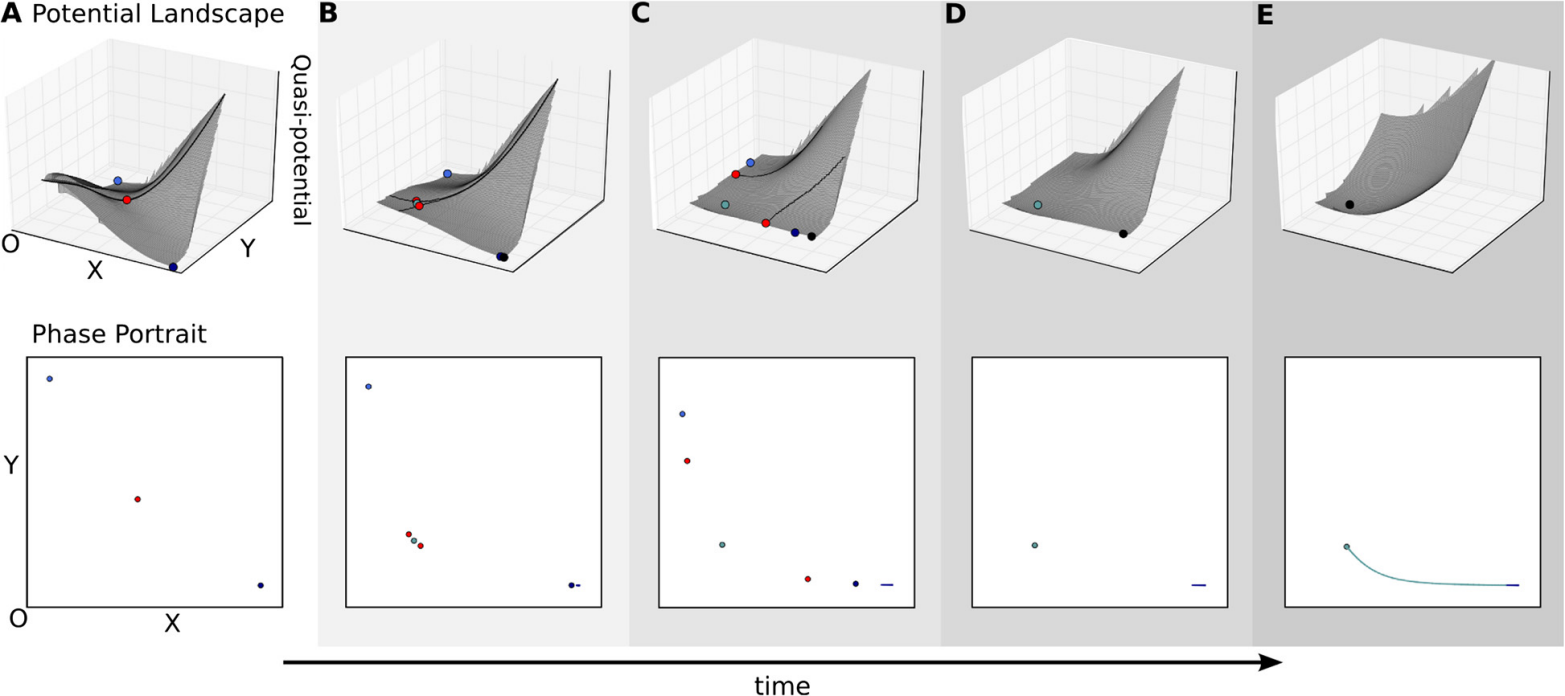
**Transition.** A transition indicates the switch of a non-autonomous system from one attractor state to another. Upper panels show (quasi-)potential surfaces, lower panels phase portraits as in Figure 3C. The progress of time is shown through increasingly dark shading, and by the arrow at the bottom of the figure. **(A)** The system starts off in the bistable regime. The trajectory’s initial conditions coincide with the attractor at high *x*, low *y* (dark blue). The trajectory is therefore at steady state at the outset. **(B)** Changes in auto-activation thresholds *a* and *c* (equation 1) over time cause the system to undergo a subcritical pitchfork bifurcation and enter the tristable regime (see also Figure 2). **(C, D)** The trajectory does not switch attractors immediately after the bifurcation occurs. However, it does not remain anchored to its current attractor either. Instead, it is left behind by the moving attractor, which it starts to pursue (see also Figures 5 and 6). **(E)** The system enters the monostable regime as the two bistable attractors disappear via two simultaneous saddle-node (or fold) bifurcations. The trajectory suddenly finds itself in an alternative basin of attraction, and eventually converges to the new, monostable attractor with low *x* and *y*. This change in basins of attraction is represented by a change in colour of the trajectory shown in (E). See also Additional file 1, Supporting Movie S1.

An example of a transition in the toggle switch model—occurring from the bistable via the tristable to the monostable regime—is shown in Figure 4 (see also Additional file 1, Supporting Movie S1). This transition is driven by an increase in the value of the auto-activation thresholds (*a* and *c* in equation 1). Changes in auto-activation have been previously used to explain the dynamics of stem cell differentiation [20,21,47,48], and patterning by lateral inhibition [25].

**Figure 5:**
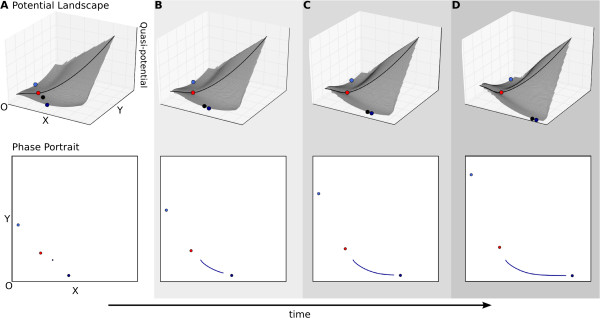
**Pursuit stabilising the direction of a trajectory.** Pursuit behaviour results from the movement of an attractor. In this case, both attractor and trajectory progress in the same direction, which is therefore stabilised even before the system enters any asymptotic regime. Upper panels show (quasi-)potential surfaces, lower panels phase portraits as in Figure 3C. The progress of time is shown through increasingly dark shading, and by the arrow at the bottom of the figure. **(A)** Pursuit behaviour shows similarities to autonomous dynamics, since the location of the initial condition determines the attractor towards which the system will converge. In this example, parameter changes do not alter the position of the separatrix. **(B–D)** However, the approach of the system towards the attractor is very different than in the autonomous case, since both attractors move away from the origin as activation strength (represented by *α*_*x*_ and *α*_*y*_ in equation 1) is increased over time. This leads to an enduring pursuit by the trajectory of its moving attractor target. See also Additional file 2, Supporting Movie S2.

**Figure 6:**
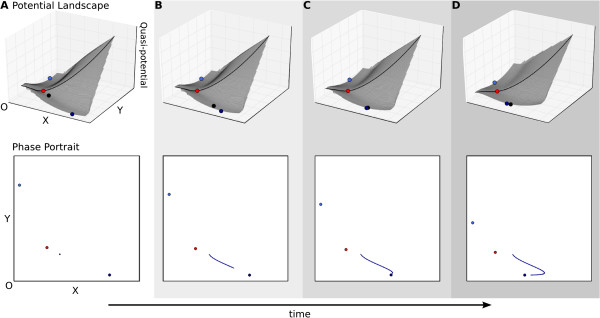
**Pursuit altering the direction of a trajectory.** Pursuit behaviour results from the movement of an attractor. In this case, the direction of the trajectory is altered since the attractor moves against the flow. Upper panels show (quasi-)potential surfaces, lower panels phase portraits as in Figure 3C. The progress of time is shown through increasingly dark shading, and by the arrow at the bottom of the figure. **(A–D)** Panels show the movement of the attractors towards the origin as activation strength (represented by *α*_*x*_ and *α*_*y*_ in equation 1) is decreased over time. As the attractor ‘overtakes’ the trajectory (between panels **B** and **C**), the angle of the flow changes drastically, leading to a reversal in the direction of the trajectory (clearly visible in **D**). See also Additional file 3, Supporting Movie S3.

In this case, we consider that the system is already at steady state at the outset, in its bistable regime with high *x* and low *y* (Figure 4A). In other words, we assume that the system has had time to converge, or that the initial condition coincides with the neighbourhood of this attractor. During the transition, two bifurcation events take place—a subcritical pitchfork bifurcation (Figure 4A to B), and two simultaneous saddle-node bifurcations (Figure 4D to E)—which make the initial steady state and its associated basin of attraction disappear while creating a new attractor at which both factors *X* and *Y* are present at low concentrations (Figure 4B to E).

Examining transient dynamics during the transition reveals important details that shape the trajectory and hence the behaviour of the system. First, we note that the trajectory initially at steady state does not necessarily remain anchored to its attractor (Figure 4C and D). As the change in parameter values causes the attractor state to move, the trajectory’s current state falls behind and reacts by travelling towards the moving attractor (see Pursuit below). Since the flow rate along the trajectory is smaller than the velocity of attractor movement, the system is not able to catch up with the moving steady state. Hence, it temporally reverts from asymptotic to transient behaviour. Obviously, such a reversal can never be observed if we only focus on steady state behaviour.

Second, we observe a delay between the subcritical pitchfork bifurcation creating the new attractor state (shown in light blue in Figure 4B) and the system switching into that basin of attraction (Figure 4E). This delay effect was already noted and described by Thom in his analysis [8]. The change from one basin to another (indicated by a change from dark to light blue on the trajectory shown in Figure 4E) only occurs once the tristable system undergoes two simultaneous saddle-node bifurcations, where the two saddles on the separatrices collide against the two outer attractors and annihilate each other (darker blue attractors in Figure 4D and E). A monostable system results from these bifurcations. The trajectory suddenly finds itself in a different basin, and eventually it will converge to this new attractor state (Figure 4E).

In this particular example, the shape of the observed transient trajectory does not differ much from its equivalent in an autonomous monostable system. This is because for most of the time the trajectory is in the basin of attraction of the attractor at low X and Y concentrations (light blue part of the trajectory in Figure 4E). Nevertheless, most developmental processes require tightly controlled timing and therefore the observed temporary reversal to transient behaviour and consequent delay in attractor switch can be significant. These aspects are therefore not negligible if we want to achieve a full understanding of the dynamics of the system. Steady state analyses, where more or less instant convergence to the new attractor state is assumed, will be limited in this regard since they are not able to address the question of developmental timing.

In addition to its effect on timing, the delay in switching basins of attraction also provides an explanation for the irreversibility of developmental pathways. This phenomenon has been studied in detail in the context of stem cell differentiation. Wang and colleagues [19,20] use a non-autonomous toggle switch model similar to ours to study the transition from monostable (stem cell) to bistable behaviour (differentiated cells). The authors simulate this transition in both directions (from stem cell to differentiated state and back), and show that two different delay effects occur depending on the direction of the process. In other words, forward and backward pathways are very different, thus explaining why the system is unable to retrace its original differentiation pathway when the process is reversed.

### Pursuit

Transient phenomena need to be taken into account to be able to fully understand the dynamical repertoire of a system. This becomes especially relevant when considering what we call pursuit mechanisms. We now consider what happens if the external activation on genes *X* and *Y* in our toggle switch model (*α*_*x*_ and *α*_*y*_ in 1) are altered over time. In biological terms, this could represent the system’s response to a changing external signal. This particular parameter change affects the geometry but not the topology of the phase portrait. The system remains in the bistable dynamical regime throughout the whole parameter range that we explored.

Let us first consider an increase in activation strength. As external production rates increase over time, the attractors move away from the origin along the direction of the *x* and *y* axes respectively (Figure 5, see also Additional file 2, Supporting Movie S2). This outward movement of the attractors leaves the position of the separatrix unchanged throughout the simulation, keeping the location and area of the basins of attraction constant over time (Figure 5). Since the separatrix does not move, trajectories are not able to change basins. Therefore, the initial conditions determine which basin of attraction the system will remain in, as they do in autonomous systems. However, the approach to the attractor is very different in this non-autonomous case, since trajectories are drawn towards a moving target. It is this continued pursuit that plays the main role in shaping them (Figure 5). Even though its associated attractor keeps moving over time, this sort of pursuit shows how the general direction of a trajectory can be stably maintained in a non-autonomous system.

Two specific pursuit scenarios can be distinguished. In the first, the trajectory eventually reaches the moving attractor. This occurs if the change in parameter values increases the flux around the attractor faster than it moves its position, or if further change in parameter values no longer alters the position of an attractor—in other words, the position of an attractor itself converges to a given location over time. Under these conditions, the trajectories of a non-autonomous system can show asymptotic behaviour and come to rest at steady state. However, it is not guaranteed that this should take place. There are many imaginable scenarios in which attractors will keep moving as parameters change and trajectories governed by pursuit mechanisms may never come to rest. It is probable that many developmental processes—at least to a certain degree—work in this regime, as external conditions and the cellular environment (signalling inputs or tissue context) constantly keep changing over time.

Just as in the case of transitions, it is obvious that pursuit mechanisms have a great impact on the timing of developmental dynamics. They can delay or even prevent the system from reaching steady state. In addition, attractor movements can also drastically influence and alter the shape of a trajectory. This becomes evident if we consider an alternative scenario, in which activating inputs decrease over time (Figure 6, see also Additional file 3, Supporting Movie S3). In this case, the attractors move towards the origin over time. This causes the attractor to ‘overtake’ the trajectory at a given point in time (Figure 6B), which induces a rather drastic change in the trajectory’s direction: initially it is travelling toward high *x* (Figure 6A), but later comes to approach the origin (low *x*) while pursuing the moving attractor (Figure 6B–D).

In summary, we have illustrated how pursuit mechanisms in non-autonomous systems can either lead to the stable maintenance of a transient trajectory (as in Figure 5), or to a drastic change in its direction (as in Figure 6) depending on the geometric arrangement of the attractor with regard to the converging trajectory. We have argued that pursuit mechanisms are likely to be almost ubiquitous in systems that are exposed to a changing environment or variable tissue context (e. g. signalling or external input by morphogen gradients). One specific example for pattern formation by pursuit are the transient gap domain shifts examined by Manu and colleagues [35,89]. In this particular case, spatial shifts in domain position are caused by the timed up- and later down-regulation of a specific sequence of gap genes in nuclei located within the posterior region of the *Drosophila* blastoderm embryo (see [35], for details). Basically, each nucleus goes through a stereotypical series of expression pulses of different gap genes. Such pulses can easily be explained by the sort of trajectory observed in Figure 6, where an initial expression trend (upregulation of *X*) is later smoothly reversed (towards downregulation of the same gene).

### Capture

In autonomous dynamical systems, trajectories never cross a separatrix [32]. Depending on the position of its initial conditions, a trajectory will find itself in a particular basin of attraction, that is, in the dynamical regime of a particular attractor. The trajectory will always remain in this basin and, given enough time, will eventually converge to the attractor.

The same is not true for non-autonomous systems. We know that the phase portrait is changing as the parameters change. This means that the position and number of steady states need not remain constant over time. We have seen how saddles and attractors can be created and annihilated by bifurcations, and how they can move through phase space in such systems. In particular, when saddle points change their position, they cause their associated separatrices to shift with them. Separatrices mark the boundaries between basins of attraction, and their movement will cause the size, shape and/or relative placement of these basins with regard to each other to change.

As trajectories progress on the changing phase portrait, it is possible for a moving separatrix to overtake them. We will call this event a ‘capture’. When a capture occurs, the affected trajectory is deviated, and often exhibits a sudden change in direction, since it is now attracted towards a different attractor at a different position of the phase plane than it was before. Captures will happen when the flow along the trajectory is smaller than the rate of change in separatrix position. The separatrix is recruiting points from one basin of attraction into another at a faster rate than the trajectory is travelling away from it. Therefore, the velocities of the trajectory and the separatrix relative to each other are determining whether a capture event is going to take place or not.

We identified two main ways by which capture events are brought about in the non-autonomous toggle switch model. In both cases, the mechanism shaping the trajectory is essentially the same: the trajectory suddenly finds itself converging towards a different attractor as a result of an abrupt change to the flow at its current position. The main difference between the two capture mechanisms resides in how the movement of the separatrix is caused.

**Figure 7:**
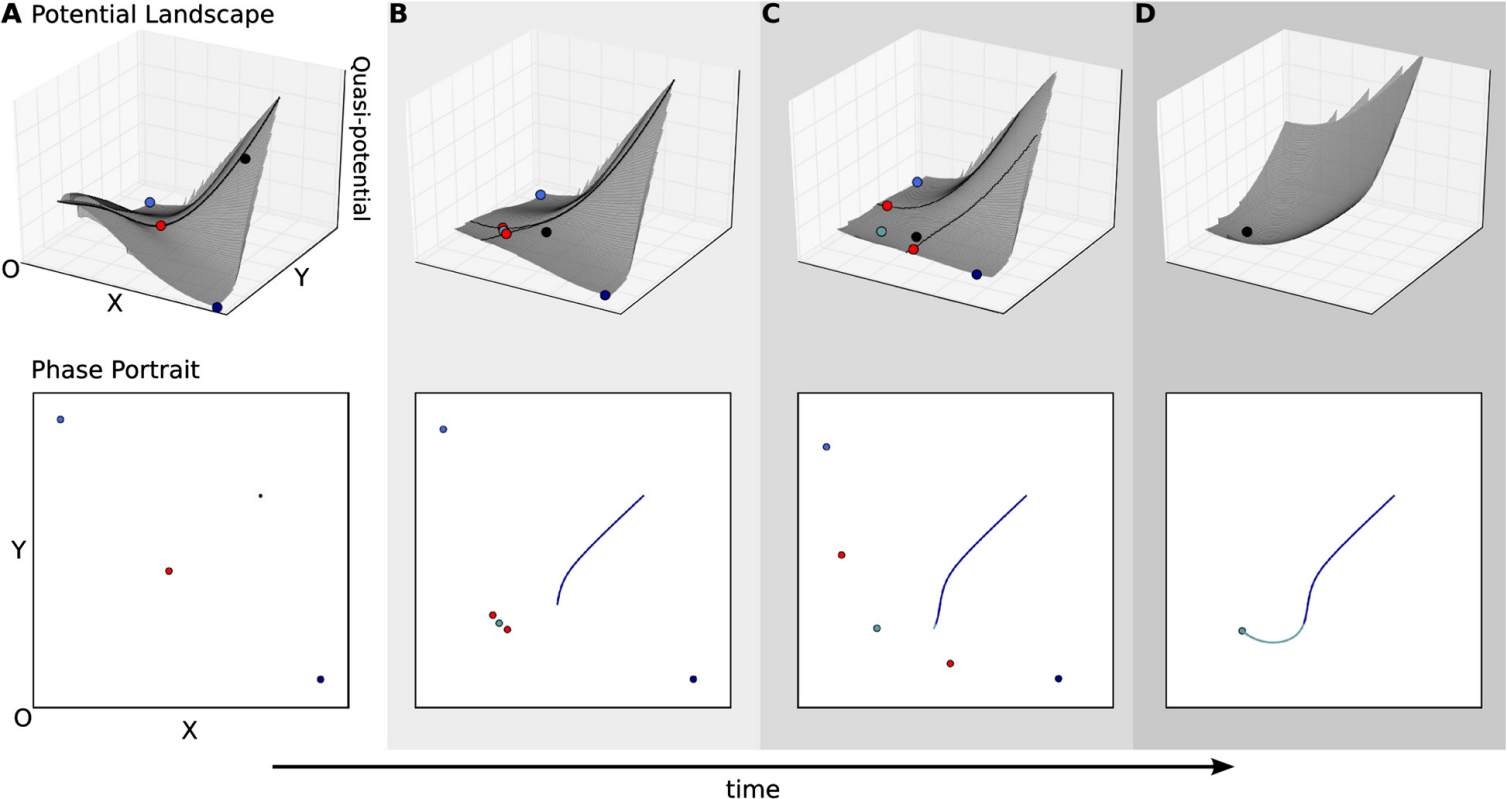
**Capture due to a change in the topology of the phase portrait.** A capture results from a trajectory being recruited into a new basin of attraction due to the movement of a separatrix. In this example, the relevant separatrix is created and caused to move by a preceding bifurcation event, which leads to the appearance of a new attractor state, resulting in a change of phase space topology. Upper panels show (quasi-)potential surfaces, lower panels phase portraits as in Figure 3C. The progress of time is shown through increasingly dark shading, and by the arrow at the bottom of the figure. **(A)** The system starts off in the bistable regime and the initial conditions place the trajectory in the basin of the high *x*, low *y* attractor (dark blue). **(B)** Changes in the values of the auto-activation thresholds (*a* and *c*, see equation 1) cause the system to undergo a subcritical pitchfork bifurcation and enter the tristable regime (see also Figure 2). At the time of the bifurcation, the trajectory is still attracted towards the dark blue attractor. **(C)** As auto-activation thresholds are further increased, the two separatrices surrounding the new attractor state (shown in light blue) separate from each other, enlarging the corresponding basin of attraction. A capture event takes place as the separatrix ‘overtakes’ the trajectory, recruiting it into the new basin of attraction. The system will now converge towards the light blue attractor. This change in basins of attraction is represented by the colour coding of the trajectory on the phase portrait. **(D)** As auto-activation thresholds are further increased, the system will transition from the tristable into the monostable regime (see also Figures 2 and 4). This causes the dark blue attractors and their basins to disappear altogether, but does not influence the direction of the trajectory anymore, which will eventually converge to the light blue attractor at low *x* and *y*. See also Additional file 4, Supporting Movie S4.

In the first situation, the trajectory gets captured after a bifurcation event has lead to the creation of a new attractor state. This increases the number of attractor basins and, in this way, introduces new separatrices into the phase portrait. We simulate this situation using the bistable-to-tristable transition caused by an increase in the auto-activation threshold as described above (Figure 7, see also Additional file 4, Supporting Movie S4). In this example, a subcritical pitchfork bifurcation creates a new attractor and two associated saddles from a pre-existing saddle point (Figure 7A,B). This results in a change in phase space topology. What used to be a single separatrix now ‘opens up’, giving rise to two different forked separatrices (Figure 7B,C). Further parameter changes then cause the new separatrices to move outward through phase space, catching up with, and overtaking, trajectories as they recruit points into the newly created and expanding basin of attraction (Figure 7B–D).

As with the transition mechanism, we notice a sometimes considerable time delay between the bifurcation and the capture event. The extent of this delay depends on how close a trajectory is to the new attractor at the time of bifurcation, and on the rate of change in separatrix position due to further parameter change. In our example, the delay effect can be clearly seen, as the trajectory first veers to the left, towards its original attractor, before being steered back towards the center of the phase portrait, where the new attractor lies. This introduces a marked bend into the trajectory (Figure 7D), which affects both time to convergence and the identity of transient states.

**Figure 8:**
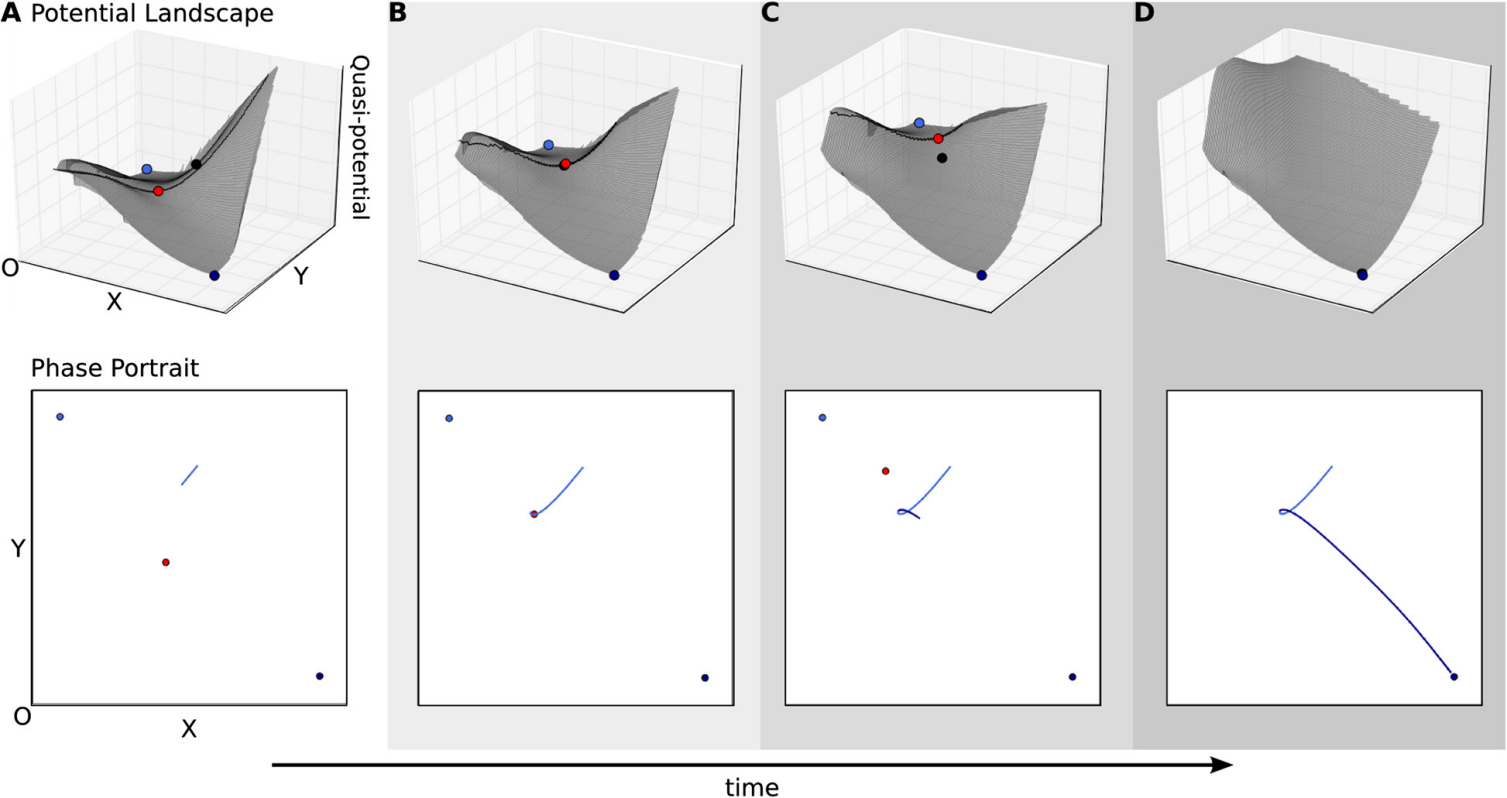
**Capture due to a change in the geometry of the phase portrait.** A capture results from a trajectory being recruited into a new basin of attraction due to the movement of a separatrix. In this example, the relevant separatrix is caused to move by a break in the symmetry of the repressive interactions between *X* and *Y*. No new attractor states or separatrices are created. Therefore, the change in landscape topography is purely geometrical and does not affect the topology of phase space. Upper panels show (quasi-)potential surfaces, lower panels phase portraits as in Figure 3C. The progress of time is shown through increasingly dark shading, and by the arrow at the bottom of the figure. **(A)** The system starts off in the bistable regime and the initial conditions place the trajectory in the basin of the low *x*, high *y* attractor (light blue). **(B)** Changing the threshold of one of the repressive interactions only (*b* in equation 1) shrinks the basin of attraction of the low *x*, high *y* attractor (light blue) causing the separatrix to move towards the upper left corner of the phase portrait. **(C)** The shifting separatrix catches up with the converging trajectory, recruiting it into the basin of the high *x*, low *y* attractor (dark blue). A capture event has taken place without any preceding bifurcation. This change in basins of attraction is represented by the colour coding of the trajectory on the phase portrait. **(D)** After the capture, the system converges to its new attractor (dark blue). Interestingly, the geometry of this capture has made the trajectory loop over itself. Such self-crossing trajectories are never observed in autonomous dynamical systems. The subsequent saddle-node bifurcation by which the saddle (red) and the light blue attractor annihilate each other does not affect the trajectory any further. See also Additional file 5, Supporting Movie S5.

In the second scenario, we consider a situation where the capture event is not preceded by a bifurcation. In this case, a pre-existing separatrix is moving through phase space due to parameter changes. This movement of the separatrix reconfigures the geometry of the basins of attraction without changing the topology of the phase portrait. We simulate this event in our toggle switch model by introducing asymmetric changes in the values of the thresholds determining the two mutually repressing regulatory interactions (i.e. *b*≠*d* in 1) (Figure 8, see also Additional file 5, Supporting Movie S5). This shifts the position of the separatrix in the bistable regime towards one of the two attractors without creating or annihilating any steady states.

As in the case of capture with preceding bifurcation, the separatrix moves and recruits points from one basin into the other, thereby shrinking one basin of attraction while enlarging the other (Figure 8A–D). If the moving separatrix encounters a trajectory at one of the points that are being recruited into a different basin of attraction, a capture event will take place (Figure 8B). The affected trajectory will change direction as it now converges towards a different attractor than before the capture (Figure 8C,D).

Capture mechanisms illustrate how dramatic changes in direction and velocity of trajectories do not always require changes to the topology of the underlying phase portraits. In other words, they do not necessarily have to be associated with bifurcations, and even if they are, the observed sudden change in direction does not usually coincide with the bifurcation. Instead, it can be significantly delayed. This kind of phenomenon cannot be observed if only asymptotic behaviours are considered. In that case, only transition-like events can occur since captures and pursuits crucially depend on transient dynamics.

There are several examples which illustrate how this might be important in practice. Corson and Siggia [90] considered an explicitly non-autonomous mechanism for determining vulval cell fates in their model. In their study, shifts in separatrices and captures happen instantaneously at the outset of the simulation. In other words, the initial condition of cells that are captured completely determines which basin of attraction they will find themselves in for the rest of the simulation. While this does not affect the analysis of the resulting differentiated (steady) states—as evidenced by the tight match between the model and the measured proportions of differentiated cells [90]—it could limit the model’s ability to reproduce the transient dynamics of cellular differentiation.

This becomes crucial when analysing more dynamic examples of gene expression, such as pattern formation driven by the gap gene system. In this example, it will be essential to study the interplay between changing maternal gradients and moving gap target domains. This was not really possible in the study of Manu and colleagues [35] since their model assumed the maternal gradients to remain constant. Our classification scheme of transient behaviour in non-autonomous systems should be useful to analyse such questions in more detail. For instance, capture events occurring at different times in different nuclei could explain how stable expression boundaries can be maintained in the presence of changing regulatory inputs. In contrast, moving domain boundaries in the posterior of the embryo are more likely to involve some type of pursuit mechanism (see above). Without doubt, the concepts developed in this paper will be useful to classify and characterise the boundary-forming and -shifting mechanisms in a fully non-autonomous model of this system.

## Conclusions

In this paper, we have argued that considering both transient dynamics and explicit time-dependence (non-autonomy) of dynamical systems is essential for an accurate and complete quantitative understanding of many biological processes. In particular, we have argued that these two features are important if we are to fully capture the richness and explanatory power of Waddington’s metaphor of the epigenetic landscape [3]. Waddington’s concepts of developmental chreodes, and their canalised nature due to the regulatory process of homeorhesis, apply to transient or non-autonomous behaviour, not stable steady states of a system. Moreover, Waddington’s landscape changes its shape due to genetic or environmental signals as represented by the pegs and ropes in Figure 1A.

However, it remains difficult to analyse transient dynamics in non-autonomous systems. Where rigorous analysis is possible, it is mostly limited to special cases (e.g., systems with regular external forcing, [82]). No general theory or toolkit exists to deal with more complex non-autonomous systems, or the transient phenomena they exhibit.

In this paper, we have taken a first step towards overcoming this limitation. We have used a pragmatic simulation-based approach to classify different mechanisms that affect transient trajectories in non-autonomous systems. Our study is based on a simple conceptual model—a toggle switch with time-dependent parameter values—to visualise the changing potential landscape which determines the dynamical repertoire of a regulatory circuit. We found four distinct mechanisms that affect trajectories on time-variable potential surfaces: transitions, pursuits and two kinds of captures. At this point it is impossible to say whether this list is complete. Other types of behaviour may be possible in higher-dimensional phase spaces.

Despite its potential incompleteness, we believe that our preliminary classification scheme is useful, since it makes transient dynamics in non-autonomous systems more tractable in practice. The advantages of our framework are twofold: first, it allows us to describe and categorise patterning mechanisms. Each one of the mechanisms described above has its own characteristics, both in terms of their effect on the short-term dynamics, as well as the longer-term evolutionary potential of the system. We have described specific examples of such characteristics in the Results and discussion Section. Second, it can be used as a diagnostic framework for the analysis of phase space, even in the case of higher-dimensional systems where the potential surface can no longer be visualised explicitly. It shows us, for example, that we need not necessarily find a bifurcation associated with every observed critical transition of a system. Instead, drastic changes in systems behaviour can also be caused by pursuit or geometrical capture events as described in the Results and discussion Section.

Our framework is widely applicable to the analysis of real-world regulatory networks. In fact, it has been developed in the context of our analysis of a fully non-autonomous version of the gap gene model by Manu *et al.*[35]. Our classification scheme has allowed us to categorise different mechanisms for spatial pattern formation in this system, and to identify those features of phase space responsible for the spatio-temporal dynamics of gene expression. A full analysis of this system will be published elsewhere. We hope that our work will inspire further such analyses in systems where our framework could be useful, and where the required detailed and data-driven models are currently available. Examples such as vulval determination in *C. elegans* or neural patterning in vertebrates come to mind. We have no doubt that—considering the current rate of progress in systems biology—this list will be extended considerably in the very near future, leading to a more general investigation of transient dynamics in non-autonomous biological systems.

## Competing interests

The authors declare that they have no competing interests.

## Authors’ contributions

BV, AC and JJ conceived and designed the study. BV implemented computational tools and performed computer simulations. BV, AC and JJ analysed results. BV and JJ wrote the paper. All authors read and approved the manuscript.

## Supplementary Material

Additional file 1 **Supporting Movie S1–Transition.** A transition indicates the switch of a non-autonomous system from one attractor state to another. This movie shows the simulation that Figure 4 is based on. It displays the changing potential landscape as the system transitions from the bistable to the tristable and then the monostable regime (see Figure 2) as auto-activation thresholds are increased. The corresponding phase portrait is shown in the inset on the upper right. Parameter values are displayed in the panel on the lower right. The current state of the system is shown as a black ball. Attractors are marked by blue dots, saddle nodes by red dots with associated separatrices shown as black lines. The history of the trajectory is shown on the phase portrait, colour coded according to which attractor the trajectory is converging towards at every individual step of the simulation. See main text and Figure 4 for details. Click here for file

Additional file 2 **Supporting Movie S2–Pursuit stabilising the direction of a trajectory.** Pursuit behaviour results from the movement of an attractor. In this case, both attractor and trajectory progress in the same direction, which is therefore stabilised even before the system enters any asymptotic regime. This movie shows the simulation that Figure 5 is based on. It displays the changing potential landscape as activation strength is increased. The corresponding phase portrait is shown in the inset on the upper right. Parameter values are displayed in the panel on the lower right. The current state of the system is shown as a black ball. Attractors are marked by blue dots, the saddle node by a red dot with its associated separatrix shown as a black line. The history of the trajectory is shown on the phase portrait. See main text and Figure 5 for details. Click here for file

Additional file 3 **Supporting Movie 3–Pursuit altering the direction of a trajectory.** Pursuit behaviour results from the movement of an attractor. In this case, the direction of the trajectory is altered since the attractor moves against the flow. This movie shows the simulation that Figure 6 is based on. It displays the changing potential landscape as activation strength is decreased. The corresponding phase portrait is shown in the inset on the upper right. Parameter values are displayed in the panel on the lower right. The current state of the system is shown as a black ball. Attractors are marked by blue dots, the saddle node by a red dot with its associated separatrix shown as a black line. The history of the trajectory is shown on the phase portrait. See main text and Figure 6 for details. Click here for file

Additional file 4 **Supporting Movie 4–Capture due to a change in the topology of the phase portrait.** A capture results from a trajectory being recruited into a new basin of attraction due to the movement of a separatrix. In this example, the relevant separatrix is created and caused to move by a preceding bifurcation event, which leads to the appearance of a new attractor state, resulting in a change of phase space topology. This movie shows the simulation that Figure 7 is based on. It displays the changing potential landscape as the system transitions from the bistable to the tristable and then the monostable regime (see Figure 2) as auto-activation thresholds are increased. The corresponding phase portrait is shown in the inset on the upper right. Parameter values are displayed in the panel on the lower right. The current state of the system is shown as a black ball. Attractors are marked by blue dots, saddle nodes by red dots with associated separatrices shown as black lines. The history of the trajectory is shown on the phase portrait, colour coded according to which attractor the trajectory is converging towards at every individual step of the simulation. See main text and Figure 7 for details. Click here for file

Additional file 5 **Supporting Movie 5—Capture due to a change in the geometry of the phase portrait.** A capture results from a trajectory being recruited into a new basin of attraction due to the movement of a separatrix. In this example, the relevant separatrix is caused to move by a break in the symmetry of the repressive interactions between *X* and *Y*. No new attractor states or separatrices are created. Therefore, the change in landscape topography is purely geometrical and does not affect the topology of phase space. This movie shows the simulation that Figure 8 is based on. It displays the changing potential landscape as the threshold for repression of *X* by *Y* is raised, causing the system to be increasingly asymmetrical. The corresponding phase portrait is shown in the inset on the upper right. Parameter values are displayed in the panel on the lower right. The current state of the system is shown as a black ball. Attractors are marked by blue dots, the saddle node by a red dot with its associated separatrix shown as a black line. The history of the trajectory is shown on the phase portrait, colour coded according to which attractor the trajectory is converging towards at every individual step of the simulation. See main text and Figure 8 for details. Click here for file
